# Bowel perforation after liver transplantation for biliary atresia: a retrospective study of care in the transition from children to adulthood

**DOI:** 10.1007/s00383-016-4008-9

**Published:** 2016-11-23

**Authors:** Yusuke Yanagi, Toshiharu Matsuura, Makoto Hayashida, Yoshiaki Takahashi, Koichiro Yoshimaru, Genshirou Esumi, Tomoaki Taguchi

**Affiliations:** Department of Pediatric Surgery, Graduate School of Medical Sciences, Kyushu University, 3-1-1 Maidashi, Higashi-ku, Fukuoka, 812-8582 Japan

**Keywords:** Bowel perforation, Liver transplantation, Biliary atresia

## Abstract

**Purpose:**

We evaluated the outcomes of liver transplantation (LT) in pediatric and adult patients with biliary atresia (BA). We focused on bowel perforation after LT (BPLT) as the most common surgical complication and analyzed its risk factors.

**Methods:**

This was a retrospective analysis of 70 BA patients who underwent LT. The patients were divided into three groups according to the timing of LT: within the first year of age (Group A), between 1 and 12 years of age (Group B), and after 12 years of age (Group C). The outcomes of LT and the clinical presentations of BPLT were compared. The surgical variables of patients with and without BPLT were analyzed to assess the risk factors.

**Results:**

The timing of LT did not affect patient survival. The incidence of BPLT was significantly higher in Group C. In Group C, BPLT progressed to severe peritonitis. No cases of BPLT-associated mortality were observed. A multivariate analysis revealed that a prolonged operative time for LT was an independent risk factor (*p* = 0.03).

**Conclusion:**

The clinical course after transplantation was complicated after adolescence. BPLT should be strongly suspected and relaparotomy should be performed in a timely manner for patients undergoing LT after adolescence.

## Introduction

Biliary atresia (BA) is the most common surgical cause of chronic cholestasis in children. If left untreated, progressive liver cirrhosis leads to death from hepatic failure, visceral bleeding and sepsis within the first years of life [1, 2]. Kasai portoenterostomy (KPE) [3] has improved the outcome of BA, particularly when it is performed during the first 90 days of life [4–6]. Some patients who attain satisfactory biliary drainage after KPE will reach adolescence without liver transplantation (LT). However, LT remains the ultimate surgery for BA and two-third of BA patients will require LT due to the progression of chronic liver disease [5, 7]. Many reports that have investigated the outcomes of LT for BA have evaluated the patients in childhood [8–11]. More than 50 years have passed since Kasai introduced KPE in 1959 [3]; thus in some cases, patients reached adulthood with their native liver [12, 13]. However, the outcomes of performing LT for BA posterior to childhood have not well discussed [14–16].

Complications after LT are common and result in significant mortality among BA patients [8–11]. Bowel perforation is associated with high rates of morbidity and mortality (2.5–20 and 30–50%, respectively) [17]. Previous studies reported that the risk factors for bowel perforation after liver transplantation (BPLT) included the pre-LT model for end-stage liver disease (MELD) score [18], a prolonged operative time for LT (duration of hepatectomy) [17, 19], previous laparotomy [11, 19–22], young age [23, 24], intra-abdominal bleeding requiring relaparotomy, early portal vein thrombosis[19], the use of high doses of steroids in immunosuppressive therapy [25] and cytomegalovirus infection [26].

We evaluated the outcomes of LT in pediatric and adult BA patients and then focused on BPLT as the most common surgical complication after LT for BA in our department. The incidence, clinical presentations, risk factors, and outcomes of BPLT after LT for BA were investigated.

## Patients and methods

Among the 92 LTs performed in 90 recipients at the Department of Pediatric Surgery in Kyushu University from October 1996 to February 2015, 70 (76.1%) were performed for BA. The other etiologies of liver failure included fulminant hepatic failure (*n* = 8; 8.7%), hepatoblastoma (*n* = 3; 3.3%), Alagille syndrome (*n* = 2; 2.2%), congenital absence of the portal vein and liver graft failure (*n* = 2; 2.2%) and one case (1.1%) each of Wilson’s disease, Primary sclerosing cholangitis, carbamoyl phosphate synthetase I deficiency, citrullinemia and hepatic failure due to familial hemophagocytic lymphohistiocytosis.

We performed a retrospective analysis of the 70 pediatric and adult BA patients who underwent LT at our department. The records were examined for the details of BPLT, the patient’s clinical status before and after LT, and the surgical variables that were possibly associated with BPLT.

The patients were divided into three groups according to the age at LT. The patients who required LT within the first year of life were classified into Group A. Then the patients who required LT later were divided at 12 years of age according to the categorization of the score for end-stage liver disease. The patients who required LT at between 1 and 12 years of age and after 12 years of age were classified into Groups B and C, respectively. First, the surgical variables and outcomes of LT were compared among the three groups. Next, the details of BPLT were analyzed among three groups. Finally, the clinical status of the patients before and after LT and the surgical variables that were possibly associated with bowel perforation after LT were analyzed in patients with and without BPLT to identify the surgical risk factors. We defined the duration between skin incision and removal of recipient’s native liver as the duration of hepatectomy. The severity of liver disease was determined using the pediatric end-stage liver disease (PELD) score in groups A and B and the MELD score in Group C.

LTs were performed under the approval from the Ethics and Indications Committee of Kyushu University. During LT, biliary reconstruction was performed using Roux-en Y hepaticojejunostomy in all cases. In all cases, immunosuppression was achieved using steroids and calcineurin inhibitors (tacrolimus or cyclosporine). Tacrolimus and cyclosporine were started at a dosage of 0.1 mg/kg/day and 5 mg/kg/day, respectively, and were adjusted based on the trough level. Methylprednisolone was progressively tapered from 3 mg/kg/day at day 1 to 0.75 mg/kg/day at day 10, and 0.3 mg/kg/day at day 30. Acute rejection was treated with steroid pulse therapy (methylprednisolone 10–20 mg/kg/day for 3 days). All of the patients received intravenous ceftriaxone or tazobactam/piperacillin for 7 days for bacterial prophylaxis and micafungin for 14 days for fungal prophylaxis.

The data are expressed as the median and interquartile range (IQR). All statistical analyses were performed using the JMP^®^ 11 software program (SAS Institute Inc., Cary, NC, USA). The comparisons among three groups were performed using Tukey’s wholly significant difference (WSD) test. The Kruskal–Wallis test was used to analyze the variance among the three groups. The Steel–Dwass test was used for nonparametric multiple comparisons of data among the three groups. Mann–Whitney‘s *U* test and Fisher’s exact test were used for the univariate analyses. A logistic regression model was used to perform a multivariate analysis. In the multivariate analysis, a variance inflation factor (VIF) of >5 was applied to exclude multicollinearity. *p* values of <0.05 were considered to indicate statistical significance and *p* values of <0.1 were considered to indicate moderate significance.

## Results

Sixty-eight living donor and two deceased donor LTs were performed for 70 BA patients. The ages of the patients at LT ranged from 5 months to 33 years (median 4.6 years).

### The patient characteristics and the outcomes of liver transplantation

The patient characteristics and the surgical outcomes were assessed according to the period of life. There were no statistically significant differences in the numbers of patients in each group (Groups A, B and C). Table 1 shows the patient characteristics and the surgical outcomes of Groups A, B and C. Factors that were significant on the Kruskal–Wallis test (*p* < 0.05) were subjected to the Steel–Dwass test; the results are summarized in Table 2. The body weight at LT increased significantly with an increase in age. The number of previous laparotomies in Group C was significantly higher than that in Group A. Six cases involved pulmonary complications. The incidence of pulmonary complications in the BA patients increased as their age increased. The PELD or MELD scores of the patients in Group A were significantly higher in comparison to Groups B and C. There was a significant decrease in the ratio of graft volume (GV)/standard liver volume (SLV) as the patients’ age category increased. The operative time for LT and the duration of hepatectomy were significantly longer in Group C. No significant differences were observed in the cold ischemic time, the duration of portal clamping or the blood loss volume.

**Table 1:** The patient characteristics and the surgical variables in the different age groups

	Group A	Group B	Group C	*p* value
Number of patients	23	23	24	
Age at LT (years)	0.8 (0.6–0.9)	4.2 (1.8–7.4)	20.5 (13.7–25.6)	<0.01
Body weight at LT (kg)	6.3 (6.1–7.4)	15.0 (9.8–20.6)	50.4 (44.5–59.6)	<0.01
Previous laparotomy (times)	1.0 (1.0–1.0)	1.0 (1.0–2.0)	2.0 (1.0–2.0)	<0.01
PELD or MELD score	19 (14–19)	5.5 (−1.5 to 16.3)	10.0 (8.0–18.0)	<0.01
Hepatopulmonary precomplications	*n* = 0	*n* = 2	*n* = 4	0.15
Type of graft	Rd-LLS (*n* = 5)	Rd-LLS (*n* = 1)	LLS (*n* = 2)	
	LLS (*n* = 17)	LLS (*n* = 16)	Left (*n* = 11)	
	Left (*n* = 1)	Left (*n* = 6)	Right (*n* = 9)	
			Whole (*n* = 2)	
GV/SLV (%)	94.6 (82.9–101.9)	68.2 (52.0–79.5)	44.6 (36.5–49.2)	<0.01
Operative time for LT (h:min)	12:22 (10:51–15:15)	12:55 (10:39–14:28)	15:17 (12:56–18:46)	<0.05
Duration of hepatectomy (h:min)	3:43 (3:15–4:33)	4:48 (4:02–5:33)	6:32 (5:31–7:42)	<0.01
Cold ischemic time (h:min)	1:50 (1:32–2:32)	1:04 (0:47–2:57)	1:59 (1:16–3:22)	<0.05
Duration of portal clamping (h:min)	2:34 (1:59–3:17)	1:53 (1:31–2:35)	1:44 (1:06–3:12)	0.15
Blood loss volume (ml/kg)	78.3 (50.6–98.9)	50.5 (39.6–148.1)	107.1 (51.9–148.4)	0.47
Posttransplant complications	BP (*n* = 4)	BP (*n* = 1)	BP (*n* = 8)	0.04
	PVT (*n* = 2)	PVT (*n* = 3)	PVT (*n* = 2)	NS
	Biliary stricture (*n* = 2)	Biliary stricture (*n* = 1)	Biliary stricture (*n* = 2)	NS
	Intra-abdominal	Intra-abdominal		
	hemorrhage (*n* = 2)	hemorrhage (*n* = 1)	–	NS
	HAT (*n* = 2)	–	HAT (*n* = 1), HAA (*n* = 1)	NS
	HVS (*n* = 1)	HVS (*n* = 1)	–	NS
	Ileus (*n* = 1)	–	–	NS
Duration of hospitalization after LT (days)	39 (28–94)	55 (50–71)	48.5 (30.6–61.8)	0.06

The data are expressed as the median and interquartile range: median (IQR)

Group A: The patients who required LT within the first year of life. Group B: The patients who required LT at between 1 and 12 years of age. Group C: The patients who required LT at after 12 years of age

*LT* liver transplantation, *PELD* pediatric end-stage liver disease, *MELD* model for end-stage liver disease, *GV/SLV* the graft volume/standard liver volume ratio, *LLS* left lateral segment, *Rd*-*LLS* reduced left lateral segment, *BP* bowel perforation, *PVT* portal vein thrombosis, *HAT* hepatic artery thrombosis, *HAA* hepatic artery aneurysm, *HVS* hepatic vein stenosis, *NS* not significant

**Table 2:** The outcomes of the nonparametric multiple comparisons among the three groups using the Steel–Dwass test

	A–B	B–C	C–A
Age at LT	*p* < 0.01	*p* < 0.01	*p* < 0.01
Body weight at LT	*p* < 0.01	*p* < 0.01	*p* < 0.01
Previous laparotomy	*p* = 0.37	*p* = 0.12	*p* < 0.01
PELD or MELD score	*p* < 0.01	*p* = 0.14	*p* < 0.01
GV/SLV	*p* < 0.01	*p* < 0.01	*p* < 0.01
Operative time for LT	*p* = 0.90	*p* < 0.05	*p* < 0.05
Duration of hepatectomy	*p* < 0.05	*p* < 0.01	*p* < 0.01
Cold ischemic time	*p* < 0.05	*p* < 0.1	*p* = 0.94

A–B: The comparison between Groups A and B. B–C: The comparison between Groups B and C. C–A: The comparison between Group C and A

*LT* liver transplantation, *PELD* pediatric end-stage liver disease, *MELD* model for end-stage liver disease, *GV/SLV* the graft volume/standard liver volume ratio

**Fig. 1:**
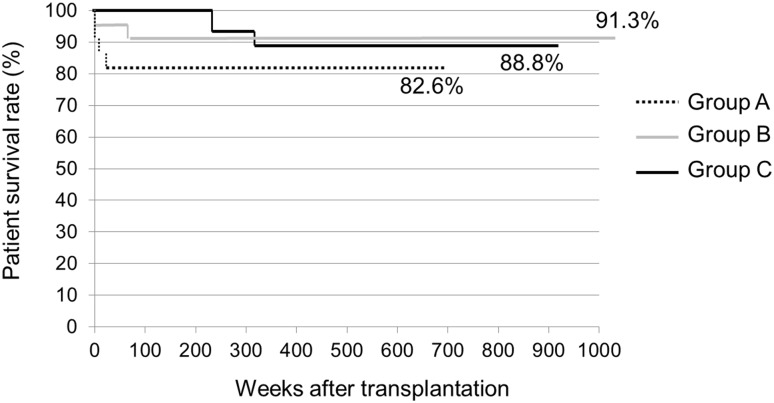
The Kaplan–Meier curves for the survival rates of patients in Groups A, B and C. Group A: The patients who required LT within the first year of age. Group B: The patients who required LT at between 1 and 12 years of age. Group C: The patients who required LT at after 12 years of age

A patient suffered from bowel perforation after LT (BPLT) due to posttransplantation lymphoproliferative disorder (PTLD) [27]. The patient was a 10-month-old girl who suffered two bowel perforations at the ileum and the transverse colon on days 94 and 394 after transplantation, respectively. To discuss the early surgical complications after LT, we excluded this case. Bowel perforation (*n* = 13) was the most common posttransplantation complication to require surgical treatment, followed by portal vein complications (*n* = 7), bile duct complications (*n* = 5), hepatic artery complications (*n* = 4), intra-abdominal hemorrhage requiring relaparotomy (*n* = 3), hepatic vein complications (*n* = 2), and intestinal obstruction (*n* = 1). Groups A, B and C included 4 (18.2%), 1 (4.3%) and 6 (24.0%) patients with BPLT, respectively. With regard to the number of BPLT cases that required relaparotomy, since two patients in Group C suffered from bowel perforation twice, the incidence in Group C (8 perforations in 24 LTs) was significantly higher than that in Group B. There were no significant differences in the incidence rates of other surgical complications among the three groups. The duration of hospital stay after LT in Groups A, B and C was 39 (28–94) days, 55 (50–71) days and 48.5 (30.6–61.8) days, respectively (*p* = 0.06). The long-term survival did not differ to a statistically significant extent among the three groups. The Kaplan–Meier survival curves are shown in Fig. 1.

### The details of bowel perforation after LT

**Fig. 2:**
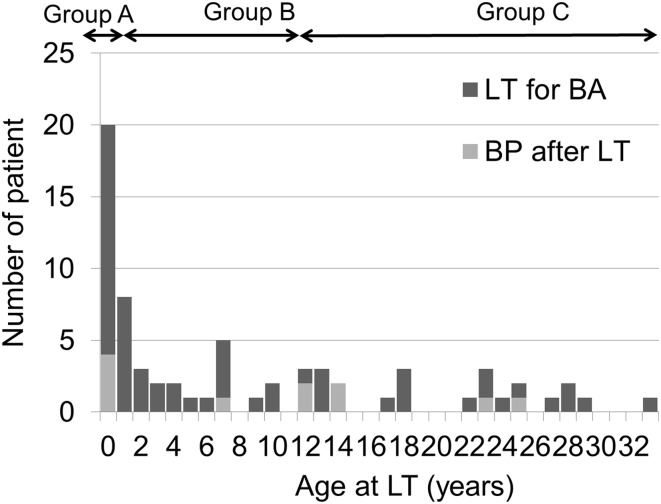
The ages of the patients who underwent liver transplantation for biliary atresia. Group A: The patients who required LT within the first year of age. Group B: The patients who required LT at between 1 and 12 years of age. Group C: The patients who required LT at after 12 years of age. *LT* liver transplantation, *BA* biliary atresia, *BP* bowel perforation

Next, we focused on BPLT as the most common surgical complication after LT for BA in our department. Excluding the case of PTLD, BPLT occurred as an early complication after LT in 15.9% of the patients (11 patients in 69 LTs). The median age of the patient with BPLT was 12.3 (0.9–14.5) years. The details of BPLT were assessed for each of the age groups. Figure 2 shows the ages of BA patients who underwent LT for BA and those who developed BPLT. Table 3 compares the characteristics of the BPLT cases among the three groups. The median time between transplantation and bowel perforation of the 11 BPLT cases was 9 (7.0–15.0) days. The median times in Groups A, B and C were 8 (7.0–13.5) days, 9 days and 11.0 (6.3–16.5) days, respectively (*p* = 0.95). The sites of perforation in Group A were localized around the liver (stomach, n = 1; duodenum, *n* = 1; jejunum, *n* = 2), while those in Groups B and C were located throughout the abdominal cavity (Roux-en Y limb, *n* = 2; ileum, *n* = 5; transverse colon, *n* = 2). Bowel perforation occurred at ten sites where adhesiotomy was performed during LT. The causes of bowel perforation at Roux-en Y limbs in two cases were ruptured sutures at the site of fixation of the Roux-en Y limb to the peritoneum. These were caused by strong traction due to stiff adhesion. The other cause in Group A was an injury to the jejunum that occurred during the placement of the nasoenteric tube in LT. One patient required steroid pulse therapy prior to BPLT due to acute rejection. One patient required relaparotomy for intestinal obstruction and steroid pulse therapy for acute rejection prior to BPLT. Cytomegalovirus infection was not observed before BPLT. Two patients in Group C had two episodes of bowel perforation. These patients had pulmonary complications associated with BA such as hepatopulmonary syndrome or pulmonary hypertension. Reperforation was observed at 2 days from the first episode in both cases; however, the site of reperforation was different from first sites.

**Table 3:** The details of the cases of bowel perforation after liver transplantation

	BPLT in Group A	BPLT in Group B	BPLT in Group C
Number of BP	4	1	8 (in 6 patients)
Time between LT and BP	8 (7–13.5) days	9 days	11.0(6.3–16.5) days
Sites of BP	Stomach (*n* = 1) Duodenum (*n* = 1) Jejunum (*n* = 2)	Roux-en-Y limb (*n* = 1)	Roux-en-Y limb (*n* = 1) Ileum (*n* = 5) Transverse colon (*n* = 2)
Clinical presentation	Abdominal distention, tachycardia	Abdominal tenderness	Abdominal tenderness
Diagnostic findings of BP	Free gas on abdominal CT	Detection of bowel contents	Detection of bowel contents
Operative procedures	Single-layer closure (*n* = 4)	Single layer closure (*n* = 1)	Single layer closure (*n* = 2) Resection and anastomosis (*n* = 3) Enterostomy (*n* = 3)

The data are expressed as the median and interquartile range: median (IQR)

Group A: The patients who required LT within the first year of age. Group B: The patients who required LT at between 1 and 12 years of age. Group C: The patients who required LT at after 12 years of age

*LT* liver transplantation, *BP* bowel perforation, *BPLT* bowel perforation after liver transplantation

The common clinical manifestations of bowel perforation in Group A were abdominal distention and tachycardia, while patients in Groups B and C most frequently presented with abdominal tenderness. Nine cases (69.2%) had fever at the time of bowel perforation. An elevated white blood cell count and serum C-reactive protein level was observed in 11 cases (84.6%), however, the patients were immunosuppressed. Most of the patients in Groups B and C were diagnosed through the detection of the bowel contents, which were obtained from an intra-abdominal drain or surgical sites. On the other hand, the abdominal fluid sampled from the intra-abdominal drains of the patients in Group A was serous due to upper gastrointestinal perforation and bowel perforation was diagnosed by the identification of free gas on abdominal CT. The operative procedures included single layer closure [*n* = 7: stomach, duodenum and ileum (*n* = 1), jejunum and Roux-en Y limb (*n* = 2)], segmental enteral resection and primary anastomosis (*n* = 3: all at the ileum) and enterostomy [*n* = 3: ileum (*n* = 1), transverse colon (*n* = 2)]. Aggressive surgery was required for the treatment of severe peritonitis due to lower gastrointestinal perforation in Group C. After the operation, all of the patients were treated with broad-spectrum antibiotics intravenously and immunosuppressive therapy was moderated; however, no patients suffered from acute rejection. All patients survived BPLT; however, two patients in Group A died from portal vein thrombosis. There was no significant difference in the duration of hospitalization after LT in the patients with and without bowel perforation [median, 50.0 (30.0–56.0) days vs. 51.0 (34.0–71.5) days, respectively; *p* = 0.37].

### The univariate analysis of the clinical status of the patients before and after LT, and the surgical variables of the patients with and without bowel perforation after LT

To analyze the surgical risk factors for BPLT, the clinical status of the patients before and after LT, and the surgical variables that were possibly associated with BPLT were compared in patients with and without bowel perforation. Table 4 shows a summary of the comparative data. The median age and body weight at LT in the patients with BPLT were 12.3 (0.9–14.5) years and 23.9 (7.5–55.2) kg, respectively. All but one patient underwent KPE as the primary operative procedure for BA. Only one patient underwent LT as the primary operative procedure for BA. The median number of previous laparotomies and PELD/MELD score were 1.0 (1.0–1.0) times and 13.0 (9.0–19.0) times, respectively. The hepatopulmonary precomplications were observed in 18.1% of the patients with BPLT and in 6.9% of the patients without BPLT, respectively. The mean ratio of graft volume (GV)/standard liver volume (SLV) in the patients with BPLT was 44.6% (36.5–88.6%). The median operative time for LT was 14 h 51 min (13 h 07 min–18 h 05 min); the median duration of hepatectomy was 4 h 38 min (4 h 07 min–6 h 27 min); the median cold ischemic time was 1 h 48 min (1 h 04 min–2 h 11 min); and the median duration of portal clamping was 2 h 05 min (1 h 08 min–2 h 49 min). The median ratio of blood loss volume/body weight in the patients with BPLT was 51.7 (29.7–141.0) ml/kg. There was a moderate significant difference in the GV/SLV ratio (*p* = 0.07) and in the operative time for LT (*p* = 0.09).

**Table 4:** The comparative analysis of the clinical status of the patients before and after liver transplantation and the surgical variables associated with bowel perforation after liver transplantation

	BPLT (+)	BPLT (−)	*p* value
Number of patients	11	58	–
Age at LT (years)	12.3 (0.9–14.5)	3.9 (1–14.5)	0.70
Body weight at LT (kg)	23.9 (7.5–55.2)	15.0 (6.7–33.2)	0.35
Previous laparotomy (times)	1.0 (1.0–1.0)	1.0 (1.0–2.0)	0.31
PELD or MELD score	13.0 (9.0–19.0)	15.0 (6.3–20.0)	0.90
Hepatopulmonary precomplications	2 (18.1%)	4 (6.9%)	0.24
GV/SLV (%)	44.6 (36.5–88.6)	72.9 (48.1–91.4)	<0.1
Operative time for LT (h:min)	14:51 (13:07–18:05)	12:59 (11:00–15:30)	<0.1
Duration of hepatectomy	4:38 (4:07–6:27)	4:49 (3:37–6:33)	0.85
Cold ischemic time (h:min)	1:48 (1:04–2:11)	1:43 (1:00–3:00)	0.67
Duration of portal clamping (h:min)	2:05 (1:08–2:49)	2:08 (1:26–3:09)	0.76
Blood loss volume (ml/kg)	51.7 (29.7–141.0)	93.7 (45.0–141.1)	0.22

The data are expressed as the median and interquartile range: median (IQR)

*BPLT* bowel perforation after liver transplantation, *LT* liver transplantation, *PELD* pediatric end-stage liver disease, *MELD* model for end-stage liver disease, *GV/SLV* the graft volume/standard liver volume ratio

### The multivariate analysis of the risk factors for bowel perforation after LT

Next, we investigated the independent risk factors for BPLT. Among the factors that were included in the univariate analyses, the body weight at LT was not included in the multivariate analysis, because the body weight at LT was highly correlated with GV/SLV and the ratio of blood loss volume/body weight (VIF >10). Both the duration of hepatectomy and portal clamping time were components of the operative time for LT; however, their VIF values were <3 and they were, therefore, included. The significant factors included a prolonged operative time for LT (*p* = 0.03). No significant difference was observed in the age at LT, previous laparotomy, PELD or MELD score, the incidence of hepatopulmonary precomplications, the GV/SLV ratio, the duration of hepatectomy, the cold ischemic time, the duration of portal clamping, or the blood loss volume (Table 5).

**Table 5:** The outcomes of the multivariate logistic regression analysis

	Odds ratio	95% confidence interval	*p* value
Age at LT	1.01	0.86–1.18	0.86
Previous laparotomy	0.68	0.12–1.94	0.49
PELD/MELD score	1.06	0.94–1.25	0.34
Hepatopulmonary precomplications	3.96	0.24–65.1	0.31
GV/SLV	0.96	0.90–1.02	0.18
Operative time for LT	1.46 (per 1-h increment)	1.03–2.21	**0.03**
Duration of hepatectomy	0.57	0.23–1.24	0.16
Cold ischemic time	0.65	0.23–1.24	0.13
Duration of portal clamping	0.55	0.15–1.41	0.24
Blood loss volume	1.00	0.98–1.00	0.63

*LT* liver transplantation, *GV/SLV* the graft volume/standard liver volume ratio

Bold value represent statistical significance (*p* < 0.05)

## Discussion

The timing of LT for BA has remained controversial. In a retrospective cohort study of 347 pediatric patients, it was reported that the patients who were underwent KPE and required LT after the first year of age showed better patient and graft survival than those who required LT within the first year of age [11]. Although some BA patients reached adulthood with their native livers, there has been limited evidence of the impact of LT on the outcome of patients who reach adulthood after KPE. Uchida et al. [15] reported that the outcome of LT in adult BA patients was significantly poorer in comparison to pediatric patients. They noted that the rates of post-transplant intestinal perforation, intra-abdominal bleeding requiring relaparotomy and biliary leakage were significantly higher in adult patients. The cumulative 5- and 10-year survival rates were 70 and 56% in adult patients, respectively. In contrast, those in pediatric patients were 87 and 81%, respectively. On the other hand, Sampedro et al. [14] and Kyoden et al. [16] reported that the outcomes of LT were satisfactory in adult BA patients. They concluded that LT can be performed safely in adult patients. In this study, with respect to the preoperative statuses of the patients, the number of previous laparotomies and the coexistence of pulmonary precomplications were higher in Group C. The PELD/MELD scores in Group A were significantly higher in comparison to Groups B and C. This is probably because the indication for LT was portal hypertension or repeated cholangitis with relatively mild liver damage. Since almost all LT patients who were treated in our department underwent living donor LT, the GV/SLV decreased as the age at LT increased. Regarding the intraoperative outcomes, the operative time for LT and the duration of hepatectomy were significantly longer in Group C. This result indicates that prolonged adhesiotomy was required due to severe intra-abdominal adhesion in Group C; thus, the incidence of BPLT was higher in Group C. However, satisfactory patient survival was achieved in all three groups.

Complications after LT are relatively common in BA patients and result in significant mortality [28, 29]. Bowel perforation is a noteworthy complication that occurs after LT in BA patients [8–11]. The incidence of this complication after pediatric LT is reported to be 6.4–20% [17, 19–23]. In the present study, bowel perforation was the most common surgical complication after LT. The incidence in the present study was relatively high (15.9%)—probably because the study population included greater numbers of adolescent and adult patients than children. Various etiologies of BPLT were reported [17–26]. One possible cause is thermal injury to the bowel during LT. In most BA patients, KPE was performed before LT and intra-abdominal adhesion was severe. Thus, we need to perform adhesiotomy carefully and gently when performing LT for BA patients. Previous studies reported that previous laparotomy and a prolonged operative time for LT were risk factors; this suggests that difficult adhesiotomy and thermal injury due to electrocautery were etiologies of BPLT. Although the number of previous laparotomies was not identified as a risk factor for BPLT in the present study, most sites of bowel perforation were observed where adhesiotomy had been performed during LT. Interestingly, the sites of perforation were observed in different locations in the patients in Group A (around the liver) and those in Groups B and C (throughout the abdominal cavity). This result indicates that KPE caused adhesion around the liver in Group A, while repeated cholangitis and multiple laparotomy caused adhesions throughout the abdominal cavity in groups B and C. Bowel injuries in the lower abdomen occurred during adhesiotomy, not only for hepatectomy but also for the construction of Roux-en Y hepaticojejunostomy in Group C. Ruptured sutures at the fixation of the Roux-en Y limb to the peritoneum due to strong traction occurred in two cases. Following these results, we usually make a Roux-en Y limb of 30 cm through the antecolic route in KPE to prevent difficult adhesiotomy and a short limb in LT. Moreover, we place adhesion barriers (Seprafilm^®^, Kaken Pharmaceutical Co., Ltd, Japan) around the liver at the end of KPE. Further studies will be necessary to establish the utility of these procedures in preventing this complication. The results of the present study suggested that Seprafilm^®^ did not have a significant effect in the prevention of BPLT (*p* = 1.0, data not shown). This result indicated that the cause of adhesion was not only KPE but also the cholangitis that occurred after the Seprafilm^®^ was resorbed. Recent studies have shown laparoscopic portoenterostomy for BA to have equivalent outcomes to open portoenterostomy [30–33]. Because laparoscopic portoenterostomy can decrease adhesion, further studies are necessary to evaluate its benefit in LT. Because laparoscopic portoenterostomy can decrease adhesion, further studies to evaluate its benefit to LT are required. Development of new surgical devices is also required to perform adhesiotomy safely and quickly.

Pulmonary complications are well recognized in chronic liver disease. The development of portal hypertension is fundamental in the pathogenesis [34]. The incidence of pulmonary complications in BA patients increased as the age of patients increased in the present study. It was reported that pulmonary complications were a risk factor for surgical complications (including infection, biliary complications, portal vein thrombosis and bowel perforation) after LT [35–37]. Multiple perforation was only observed in the patients with pulmonary complications in this study. In addition to these two cases, a patient in Group C suffered from multiple bowel perforations following splenectomy before LT. Although pulmonary complications did not show strong statistical power because of the small sample size of the present study, pulmonary complications remain a risk factor for BPLT.

In our cases, fever or the elevation of inflammatory marker levels was observed in most patients, despite the patients being immunosuppressed. Bowel perforation was suggested to have triggered the severe immune response and distressed the patients. Because the sites of perforation in Group C were localized in the lower tract, peritonitis was relatively severe, even if the diagnosis was immediate. Aggressive operations, such as segmental enteral resection and primary anastomosis or enterostomy were, therefore, indicated for most of the patients in Group C. Xiong et al. [38] reported that among adults with BPLT, the patients who had severe abdominal cavity contamination tended to die despite undergoing enterostomy. Thus, an early diagnosis may ensure better survival. While the rate of mortality due to bowel perforation after pediatric LT is reported to be 30–50% [17], we did not encounter any cases of BPLT-associated mortality in the present study. Timely laparotomy and aggressive operations seemed to prevent deaths due to this life-threatening complication in our department.

As in previous reports [17, 19], a prolonged operative time for LT was found to be an independent risk factor for BPLT in a multivariate analysis with logistic regression in the present study. This result also suggests that severe intra-abdominal adhesion is a risk factor for BPLT because the duration of adhesiotomy had a strong impact on the operative time for LT. While a prolonged operative time for LT was identified as an independent risk factor, the duration of hepatectomy did not differ to a statistically significant extent. This is also depended on the duration of adhesiotomy. A possible reason for this difference is that adhesiotomy was performed not only during hepatectomy but also during Roux-en Y hepaticojejunostomy construction. In two cases of bowel perforation, the perforation occurred due to the rapture of sutures at the site of fixation of the Roux-en Y limb to the peritoneum. Although thermal injury due to electrocautery was not the etiology, adhesiotomy for the construction of Roux-en Y hepaticojejunostomy took a long time in these two cases. Thus, a prolonged operative time for LT showed strong statistical power. In this point, we must be careful until the end of the operation, even if a longer operative time is required.

In summary, the timing of LT did not affect patient survival after LT for BA. However, the incidence of BPLT was significantly higher in patients who were older than 12 years of age. Furthermore, these patients suffered from more severe peritonitis, which required aggressive surgery. The patients who required LTt after 12 years of age showed a potentially higher risk of BPLT because they required longer operations due to severe adhesion throughout the abdominal cavity and the coexistence of pulmonary complications. Since the clinical course after transplantation was complicated after adolescence, LT should be performed, as early as possible for patients who are diagnosed with progressive liver disease after KPE. Patients who undergo LT after adolescence, should be carefully observed to allow for an immediate diagnosis of BPLT and timely laparotomy should be performed to treat this lethal complication. The population of the present study was small. Thus, further studies are necessary to clarify the optimal timing of LT for BA patients.

## Abbreviations

BA: Biliary atresia
KPE: Kasai portoenterostomy
LT: Liver transplantation
BPLT: Bowel perforation after liver transplantation
PTLD: Posttransplantation lymphoproliferative disorder
PELD: Pediatric end-stage liver disease
MELD: Model for end-stage liver disease
GV/SLV: The ratio of graft volume/standard liver volume
